# Structure-based virtual screening for selective connexin hemichannel blockers: a historical perspective on the discovery of a small organic inhibitor

**DOI:** 10.3389/fphar.2026.1870076

**Published:** 2026-06-25

**Authors:** Carlos F. Lagos, Anibal Vargas, Anibal García, Yorley Duarte, Chenju Yi, Juan C. Sáez

**Affiliations:** 1 Chemical Biology and Drug Discovery Lab, Escuela de Química y Farmacia, Facultad de Ciencias, Universidad San Sebastián, Santiago, Chile; 2 Centro Basal Ciencia & Vida, Fundación Ciencia & Vida, Santiago, Chile; 3 Center of Integrative Biology, Faculty of Science, Universidad Mayor, Santiago, Chile; 4 Instituto de Neurociencias, Centro Interdisciplinario de Neurociencias de Valparaíso, Universidad de Valparaíso, Valparaíso, Chile; 5 Center for Bioinformatics and Integrative Biology, Facultad de Ciencias de la Vida, Universidad Andres Bello, Santiago, Chile; 6 Department of Geriatrics, Seventh Affiliated Hospital of Sun Yat-sen University, Shenzhen, China; 7 Shenzhen Key Laboratory of Chinese Medicine Active Substance Screening and Translational Research, Shenzhen, China

**Keywords:** connexin, D4, gap junction, hemichannel, selective blocker, structure-based drug design, virtual screening

## Abstract

**Introduction:**

Connexin hemichannels are critical mediators of cellular signaling in both physiological and pathological states, yet their selective pharmacological modulation has remained elusive because of the close structural similarity between hemichannels and gap junction channels formed by the same connexins.

**Methods:**

We combined structure-based virtual screening of a connexin hemichannel model with experimental validation — ethidium/DAPI dye-uptake assays, single-channel and whole-cell electrophysiology, and Lucifer yellow gap-junctional coupling assays — to identify and characterise D4, a small chiral organic molecule, and we synthesised and tested both of its enantiomers.

**Results:**

D4(R) potently and selectively blocks C×43 hemichannels at nanomolar concentrations (IC_50_ ≈ 5 nM) without inhibiting gap junction channels formed by the same connexin, and it does not block C×36 hemichannels. The R-enantiomer is fully active whereas the S-enantiomer is inactive, demonstrating stereospecific molecular recognition. Electrophysiology confirms complete blockade of discrete C×43 hemichannel currents at 10 nM, with no effect on pannexin-1, CALHM1 or TRPV2 channels.

**Discussion:**

These findings establish D4(R) as a first-in-class, gap-junction-sparing hemichannel blocker and provide a validated molecular scaffold for next-generation connexin therapeutics, addressing a long-standing challenge in connexin pharmacology and opening new avenues for dissecting hemichannel function under physiological and pathophysiological conditions.

## 1 Introduction

Connexins constitute a family of transmembrane proteins that oligomerize into hexameric hemichannels (connexons) at the plasma membrane (Sosinsky and Nicholson, 2005). These hemichannels can function as standalone conduits between the cytoplasm and extracellular space or dock with hemichannels on adjacent cells to form gap junction channels (Saez et al., 2003). In their undocked state, hemichannels mediate the transmembrane transfer of metabolites, nutrients, ions, and signaling molecules including ATP, glutamate, prostaglandin E_2_, and NAD^+^, among others (Retamal et al., 2007; Sáez et al., 2010). Under physiological conditions, hemichannel activity is tightly regulated to prevent excessive membrane permeability that could lead to deleterious consequences (Sáez et al., 2025). Accordingly, pathological upregulations of hemichannel opening contributes to inflammatory responses, cell death across multiple organ systems and tissue dysfunction (Orellana et al., 2009; Lillo et al., 2019). Aberrant elevated hemichannel activity has been shown to play crucial roles in neuroinflammation, and in different pathological conditions such as cardiac arrhythmias, skeletal muscle pathologies, and skin disorders (Munoz Camus et al., 2024; Zhao et al., 2024; Mammano et al., 2025). In agreement with these findings, astrocyte and microglial hemichannel openings enable the release of ATP and glutamate in the brain, driving neuroinflammation and leading to inflammasome activation, spreading depression, and behavioral phenotypes in preclinical models (Tichauer et al., 2024; Su et al., 2025). At the molecular levels, S-nitrosylation of connexin43 (Cx43) at Cys271 activates undocked hemichannels in Duchenne muscular dystrophy cardiomyopathy, promoting arrhythmogenic membrane permeability and myocardial arrhythmia (Munoz Camus et al., 2024). Collectively, these observations position connexin hemichannels as attractive therapeutic targets for diseases characterized by pathological channel activity.

### 1.1 Structural basis for connexin channel function

**FIGURE 1:**
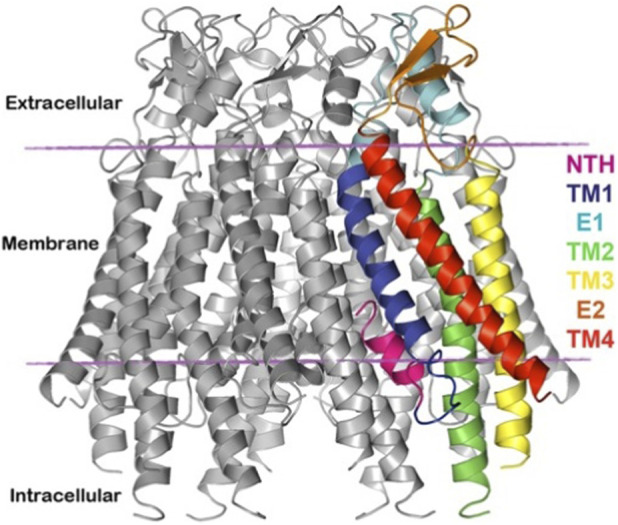
Schematic representation of the basic structural organization of connexin hemichannels. Secondary structure model of a connexin hemichannel formed by the hexameric arrangement of connexin protomers. A single protomer is highlighted to illustrate the domain architecture, with the N-terminus (NTH), transmembrane segments (TM1–TM4), extracellular loops (E1 and E2), and cytoplasmic loop (CL) shown in distinct colors. Solid purple lines indicate the approximate membrane planes separating the extracellular, membrane, and intracellular regions.

Recent structural advances have begun to illuminate the molecular basis for hemichannel gating and inhibitor recognition. Each connexin protomer contains four transmembrane α-helices (TM1–TM4), two extracellular loops (E1 and E2), a cytoplasmic loop (CL), and cytoplasmic N- and C-termini (Unger et al., 1999). The N-terminal helix (NTH) lines the pore and plays a critical role in gating and permeability (Figure 1). The crystal structure of connexin26 gap junction channels at 3.5 Å resolution revealed that hemichannels are conserved modular units, each comprising six connexin protomers arranged around a central pore (Maeda et al., 2009).

Cryo-electron microscopy (cryo-EM) studies have provided unprecedented insights into inhibitor binding and gating mechanisms. Notably, cryo-EM structures of human Cx36 revealed that inhibitors such as mefloquine, arachidonic acid, and 1-hexanol induce displacement of the pore-lining N-terminal helices from a pore-lining (PLN) state into a flexible N-terminal (FN) state, with flat lipid densities blocking the pore (Cho et al., 2024). Furthermore, cryo-EM and mutagenesis studies of Cx32 and Cx43 identified two recurring inhibitor sites: A pore-buried site M where mefloquine modifies pore electrostatics and partially occludes permeation, and a site A near the N-terminal gating helix where 2-aminoethoxydiphenyl borate (2-APB) restricts the pore entrance (Lavriha et al., 2025). These structural insights provide a rational framework for designing isoform-selective inhibitors. Additionally, the structural trapping of wild-type and K125E mutant Cx26 demonstrated that CO_2_ mimicking a negative charge at K125 favors a constricted pore entrance, linking cytoplasmic loop positions, transmembrane helix conformations, and N-terminal helix positioning to a gating mechanism regulated by carbamylation and charge (Brotherton et al., 2024). Computational modeling studies have previously suggested that the N-terminal helix region may serve as a binding site for connexin modulators such as D4(R), mefloquine and 2-APB (Guo et al., 2022; Comollo, 2018), providing a structural rationale for targeting this domain in drug discovery efforts. Collectively, these studies underscore the importance of N-terminal helix dynamics and ligand-induced conformational changes in controlling hemichannel activity.

### 1.2 Connexin hemichannel modulators

Despite the recognized importance of hemichannels in disease, pharmacological tools to selectively modulate these membrane channels have remained limited (Laird and Lampe, 2018; Natha et al., 2021; Subedi et al., 2021). In a few cases, the expressed connexin type would form hemichannels, but not functional gap junction channels, as is the case of Cx39 (Vargas et al., 2017), offering a natural opportunity to understand the role of hemichannels over that of putative gap junction channels. However, most cells express connexins that serve as a common backbone for hemichannels and gap junction channels. Therefore, antisense RNAs or morpholinos are not useful to dissect the importance of each co-expressed channel type. In addition to this limitation, functional gap junctions are essential for numerous tissue functions, and their inhibition is likely to cause undesirable side effects. The generation of inhibitors of one channel type (e.g., hemichannels) was expected to also inhibit the other channel type (e.g., gap junction channels), given that both channel types are composed of the same backbone. However, this expectation did not consider differences in their structures due to diverse interactions with other cytoplasmatic molecules. An attempt to solve this limitation was the generation of peptides that cause the rapid inhibition of hemichannels acting on extracellular domains of connexins, interacting with protein domain not exposed in gap junction channels, but causing a slower reduction in gap junction channel activity by preventing the formation of new gap junction channels (Froger et al., 2010), which might lead to erroneous result interpretations. To resolve this limitation, a more effective method consisted of generating TAT modified peptides that cross the cell membrane and block hemichannels *via* interactions with intracellular domains of connexins, such as TAT-Gap19 and TAT-Cx43_266–283_ peptides, which block hemichannels but not gap junction channels, and do not reduce the formation of gap junction channels in long term treatments (Gangoso et al., 2017). Although these peptides have been useful under experimental conditions, their effective concentrations *in vitro* are between 100 and 200 μM, indicating a low affinity for the desired molecular target, and therefore, they could interact with other molecules that have not yet been identified.

An early finding of a small molecule that blocks hemichannels without significant effects on gap junction channels corresponds to boldine (Hernández-Salinas et al., 2013). This work was inspired by the protective effect of boldine on cell-cell uncoupling induced by the tumor promoter TPA (Hu et al., 1995), but its effect on hemichannels was not studied. The IC_50_ of boldine is 50 μM, suggesting poor selectivity, and cannot clearly help answer the questions mentioned above. In line with this, later studies discovered that boldine blocked additional large-pore channels like the P2X7 receptor and pannexin1 hemichannels (Yi et al., 2017; Toro et al., 2023). Other small molecules found to block hemichannels and not gap junction channels were aminoglycoside antibiotics like gentamycin (Figueroa et al., 2014). As a follow-up, amphiphilic aminoglycosides without antibiotic effects that retained their inhibitory effect over connexin hemichannels were generated (Fiori et al., 2017; Alfindee et al., 2018). To our knowledge, their affinity, selectivity and use on animal models of human diseases have yet to be studied. At that time, there were several connexin-based channel modulators, but they simultaneously affected hemichannels and gap junction channels. The most available hemichannel blockers, including carbenoxolone, glycyrrhetinic acid derivatives, flufenamic acid, and quinine were also found to inhibit gap junction channels, making it difficult to differentiate the distinct functions of hemichannels *versus* gap junctions (Bodendiek and Raman, 2010; Acosta et al., 2021). These compounds are often poorly selective, effective only at micromolar concentrations, and can inhibit other large-pore channels such as pannexin hemichannels and the CALHM1 channel (Caterina et al., 2000; Penuela et al., 2013). Consequently, the lack of gap-junction-sparing hemichannel blockers has hindered mechanistic studies and therapeutic development.

Thus, the compounds described in the state-of-the-art displayed little efficacy and unspecific mechanisms of action or activity secondary to the pharmacological action in other therapeutic targets that modify the activity of connexin hemichannels (e.g., protein kinases and phosphatases). Hence, pharmacological tools to elucidate the functions of gap junction channels independent from those of hemichannels and to validate them as drug targets to develop novel therapies for connexin-based diseases were urgently needed.

With growing structural information about connexin channels and their limitations in terms of hemichannel blockers, we sought to identify novel small-molecule inhibitors that selectively block hemichannels without affecting gap junction channels. We used structure-based virtual screening targeting the N-terminal helix domain and transmembrane segments of connexin hemichannels, followed by rigorous experimental validation using dye uptake assays, electrophysiology, and selectivity profiling against other large-pore channels. Our goal was to describe a historical perspective of the discovery of a first-in-class gap-junction-sparing hemichannel blocker that could serve as both a research tool and a lead compound for therapeutic development.

## 2 Materials and methods

### 2.1 Structure-based virtual screening

**FIGURE 2:**
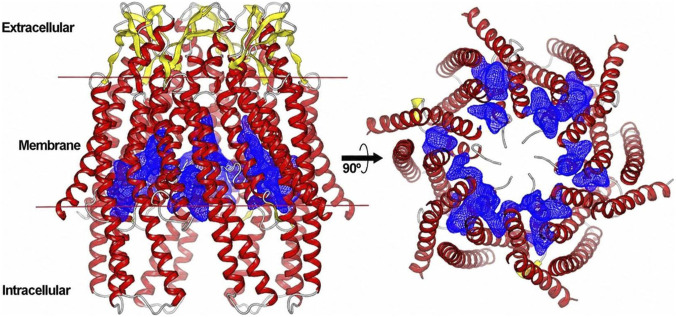
Secondary structure representation of the connexin hemichannel and predicted ligand-binding site. Side and top views of the connexin hemichannel are shown, with the putative ligand-binding site highlighted in blue. Candidate ligand poses at receptor site 100 were obtained and optimized using the Chemgauss3 scoring function. Final consensus binding modes from the docking workflow were selected by consensus scoring based on the PLP, ChemScore, and Chemgauss3 evaluation functions.

The virtual screening workflow was designed to identify small molecules capable of binding to a putative ligand-binding pocket within the connexin hemichannel structure (Figure 2). The crystal structure of the connexin26 (Cx26) gap junction channel (PDB ID: 2ZW3) was used as a structural template (Maeda et al., 2009). The hemichannel unit was extracted, and the binding site was defined based on the N-terminal helix (NTH) domain and adjacent transmembrane segments (TM1–TM4), which are critical for gating and permeability (Sosinsky and Nicholson, 2005). First, the crystalline structure of the hemichannel formed by human Cx26 was completed. Then, we prepared the comparative models founded on the hypothesis that the binding of compounds to a pocket limited by the N-terminal helical (NTH) domain, the transmembrane segments of helixes TM1 and TM2 of a protomer and the transmembrane segments of helixes TM1 of the adjacent protomer prevent the conformational changes necessary to generate the opening/activation of the hemichannels formed by connexins. A total of 500 models of hemichannels formed by human Cx26 or Cx43 were generated using Modeller (Martí-Renom et al., 2000), and the best model selected according to the internal DOPE scoring was selected for further analysis and optimization. The validation of the structures was performed by using the tools available on the web server SAVES (https://saves.mbi.ucla.edu/).

Before the virtual screening process, the database of chemical compounds OpenNCI (approx. 260,000 compounds) was filtered in order to eliminate the salts and counterions using the program FILTER v2.1 (OpenEye Scientific Software) and then converted to 3D coordinates using OMEGA v2.4.6 (OpenEye Scientific Software) (Hawkins et al., 2010), thus providing a database of about 195,000 compounds and conformers. They were subject to a comprehensive molecular coupling protocol in the modified crystalline structure of the hemichannel formed by Cx26 and the hemichannel model formed by Cx43 using the software FRED v2.2.5. The binding sites of the hemichannel formed by Cx26 and Cx43 were identified and prepared using the FRED receptor program (McGann, 2011; McGann, 2012).2The binding site was defined by the region comprising the residues of 3–10 (NTH), 29–40 (TM1), 74–93 (TM2) of a protomer and residues 29–40 (TM1) of an adjacent protomer in connexin 26 or the equivalent residues in other connexins, a region previously implicated in ligand binding through computational modeling (Cisterna et al., 2020).

The candidate binding modes of ligands at the receptor site (100) were obtained and optimized by using the Chemgauss3 scoring function. The structures of consensus for the binding modes in the comprehensive molecular coupling process and optimization were obtained through consensus scoring with the assessment functions PLP, Chemscore and Chemgauss3 (McGann et al., 2003). Finally, the binding modes for the best classified 1,000 compounds were minimized with the force field CHARMm22 in DiscoveryStudio v2.1 (Accelrys Inc., San Diego). The protocol allows for minimizing the side chains of residues inside six Å from the mass centroid of all coupled ligands, using the conjugated gradient algorithm up to a convergence criterion of 0.001 Å kcal/mol for the root mean square (RMS) of the energy gradient. Evaluation of the binding energy was performed for each complex obtained using the evaluation functions PLP, LigScore, PMF and LUDI. For the final identification of compounds, the consensus scoring protocol available in Discovery Studio v2.1 was used. From this new classification, results were visually inspected for compounds with the 100 best results, and 40 compounds were selected within the group showing the best affinity to hemichannels formed by Cx26 or Cx43.

### 2.2 Biological evaluation of hemichannel activity

Cells were seeded in black 96-well plates at a density of 1 × 10^3^ cells per well and maintained for 24 h before exposure to the putative hemichannel blockers. Cells were exposed to divalent cation-free saline solution (DCFS), which increases the open probability of hemichannels by removing extracellular Ca^2+^ and Mg^2+^ (Sáez et al., 2014) in the presence of ethidium bromide (Schalper et al., 2008). Fluorescence was quantified in cells plated on black 96 well plates (Corning, Shoreview, MN. USA) to minimize light scattering and reflection of the excitation source in HeLa tranfectants incubated 5 min in recording Krebs solution without Ca^2+^ and Mg^2+^ plus 5 µM ethidium bromide, included as permeability tracer. The total fluorescence emission due to ethidium bromide cell uptake followed by fluorescence emission upon intercalation of ethidium with nucleic acid present into each cell was evaluated using a fluorometer Perkin Elmer LS50 B fluorometer equipped with a plate reader. Thus, dye uptake measurements were obtained from individual cells rather than from the entire well. Carbenoxolone (CBX, 100 µM) or 18β-glycyrrhetinic acid (18βGA, 100 µM) was used as a positive control, whereas non-transfected HeLa cells were used as a negative control.

In time-lapse experiments, DAPI or ethidium bromide was used as permeability tracer as previously described (Contreras et al., 2003; Vargas et al., 2017; Cisterna et al., 2020). For Intracellular Ca^2+^ signal to evaluate the effect of D4 on TRPV2 channels we used FURA-2 as described previously (Cisterna et al., 2020).

### 2.3 Gap junction channel coupling assays

Gap junction channel function was evaluated using Lucifer yellow (LY) microinjection assays in confluent cultures of HeLa cells transfected with mCx43, mCx45, mCx32, or mCx36 (Contreras et al., 2002). Cells were grown to confluence to promote gap junction formation. LY (molecular weight ∼457 Da) was microinjected into individual cells using glass micropipettes, and dye transfer to neighboring cells was monitored by fluorescence microscopy. Coupling incidence was defined as the percentage of injected cells that transferred LY to at least one adjacent cell. A minimum of 20 cells were injected per condition in three independent experiments. Cells were treated with D4(R) (50 nM) or vehicle control for 30 min prior to microinjection.

### 2.4 Electrophysiological recordings

Whole-cell and single-channel patch-clamp recordings were performed on HeLa cells transfected with mCx43 to directly assess the effect of D4(R) on hemichannel currents using whole cell voltage clamp (Contreras et al., 2002). Cells were voltage-clamped at a holding potential of 0 mV, and voltage ramps from −80 mV to +80 mV were applied to elicit hemichannel currents. Divalent cation-free saline solution (DCFS) was used to increase the activity of hemichannels, and D4(R) (10 nM) was applied *via* bath perfusion. Current-voltage (I-V) relationships were constructed from voltage-ramp recordings. Single-channel recordings were obtained in cell-attached configurations, and conductance histograms were generated to quantify channel-opening events. Data were acquired using an Axopatch 200 B amplifier and analyzed with pCLAMP software.

### 2.5 Selectivity profiling against other large-pore channels

D4(R) concentrations were selected according to the experimental objective, using nanomolar ranges for potency assays and micromolar ranges for selectivity assays. To assess D4(R) selectivity, we tested its effects on pannexin 1 (Panx1), CALHM1, and TRPV2 channels. For Panx1, HeLa cells stably expressing Panx1-EGFP were subjected to mechanical stress to activate hemichannels, and EtBr uptake was measured in the presence of D4(R) (100 nM) or carbenoxolone (5 µM) (Penuela et al., 2013). For CALHM1, HeLa cells expressing CALHM1-EGFP were exposed to Ca^2+^/Mg^2+^-free solution to activate channels, and DAPI uptake was measured in the presence of D4(R) (50 µM), La^3+^ (20 µM), or ruthenium red (20 µM) (Ma, Siebert et al., 2012). TRPV2 was included in the selectivity analysis as a non-connexin Ca^2+^-permeable channel to determine whether D4 interferes nonspecifically with stimulus-evoked Ca^2+^ entry pathways. For TRPV2, HeLa cells transfected with TRPV2 were loaded with Fura-2 AM, and intracellular Ca^2+^ signals were monitored following activation with 2-APB (100 µM) in the presence or absence of D4(R) (100 nM) (Caterina et al., 2000).

### 2.6 Chemical synthesis of D4 enantiomers

Two synthetic routes were developed to prepare the R- and S-enantiomers of D4. Method I employed a dithiane-based Umpolung strategy: 1,3-dithiane was formed from 4-chlorobenzaldehyde, lithiated with n-BuLi, and subjected to nucleophilic addition to benzaldehyde. Oxidation followed by enantioselective Corey-Bakshi-Shibata (CBS) reduction afforded the chiral secondary alcohol intermediate, which underwent EDCI-mediated esterification with a heteroaromatic carboxylic acid. Final dithiane deprotection regenerated the carbonyl group, yielding D4. Method II applied a Weinreb amide strategy: an α-hydroxy acid was converted into the corresponding methyl ester and subsequently transformed into a Weinreb amide. Adding 4-chlorophenylmagnesium bromide provided the α-hydroxy ketone intermediate, which was then esterified with a heteroaromatic carboxylic acid using EDC·HCl/DMAP to afford D4. Enantiomeric purity (>99%) was confirmed by chiral HPLC, and structures were verified by ^1^H NMR, ^13^C NMR, and high-resolution mass spectrometry (HRMS).

## 3 Results

### 3.1 Virtual screening identifies candidate hemichannel modulators

**FIGURE 3:**
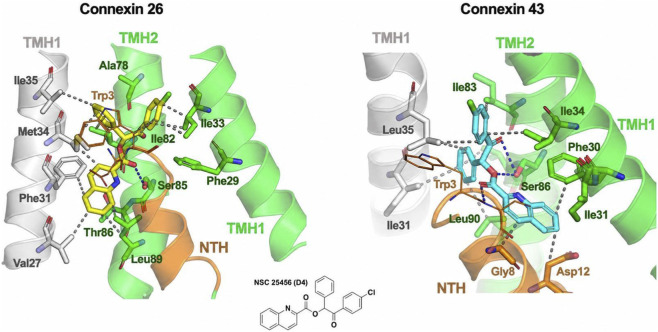
Predicted binding mode of D4 in connexin 26 and connexin 43 hemichannels. Docking poses of D4 in the putative intersubunit binding pocket of connexin hemichannels. The left panel shows D4 bound to Cx26, the center panel shows the chemical structure of D4, and the right panel shows D4 bound to Cx43. The predicted pocket is formed by residues 3–10 of the N-terminal helix (NTH), residues 29–40 of TM1, and residues 74–93 of TM2 from one protomer, together with residues 29–40 of TM1 from the adjacent protomer, or the equivalent region in other connexins. In Cx26, D4 is stabilized by hydrophobic interactions with Trp3, Val27, Phe31, Ile33, Met34, Val37, Ile82, Thr86, and Leu89, a hydrogen bond with Ser85, and a π-stacking interaction with Trp3. In Cx43, D4 forms predominantly hydrophobic contacts with Gly8, the aliphatic portion of Asp12, Phe30, Ile31, Ile34, Leu35, Ile83, Val87, and Leu90, and hydrogen bonds with Ser5 and Ser86. These predicted interactions support D4 binding within a conserved pocket that may contribute to stabilizing a non-conducting hemichannel conformation.

Structure-based virtual screening of approximately 190,000 compounds identified candidates predicted to bind a pocket located between the N-terminal helix and the transmembrane segments of connexin hemichannels. From the 40 compounds selected for experimental evaluation, D4(R) emerged as a relevant hit because it showed blocking activity against both Cx26 and Cx43. Docking analysis predicted that D4(R) binds within the same intersubunit cavity in both hemichannels (Figure 3), establishing predominantly hydrophobic interactions with residues lining the pocket. In Cx26, the interaction pattern involved Trp3, Val27, Phe31, Ile33, Met34, Val37, Ile82, Thr86, and Leu89, together with a hydrogen bond to Ser85 and a π-stacking interaction with Trp3. In Cx43, D4(R) interacted with Gly8, the aliphatic portion of Asp12, Phe30, Ile31, Ile34, Leu35, Ile83, Val87, and Leu90, and formed hydrogen bonds with Ser5 and Ser86. Overall, these results indicate that D4(R) targets a structurally related binding pocket in both connexins, providing a plausible structural basis for its dual inhibitory activity.

### 3.2 D4(R) potently inhibits connexin hemichannels with nanomolar potency

**FIGURE 4:**
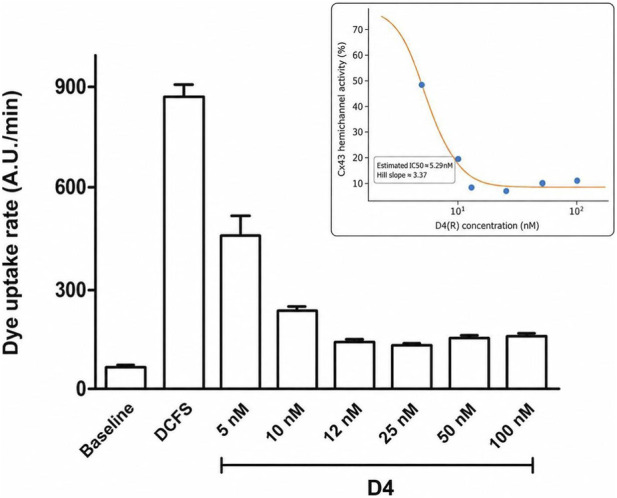
D4 inhibits Cx43 hemichannel activity in a concentration-dependent manner. Dye uptake rate (A.U./min), used as an index of Cx43 hemichannel activity, was measured under basal conditions, after divalent cation-free solution (DCFS) stimulation, and following treatment with increasing concentrations of D4(R) (5–100 nM). DCFS markedly increased ethidium bromide uptake, whereas D4(R) suppressed this effect in a concentration-dependent manner, reducing uptake to near-basal levels at concentrations of 12.5 nM and above. The inset shows the normalized concentration–response curve obtained from the same dataset and fitted using a sigmoidal dose–response model, yielding an estimated IC_50_ of 5.29 nM and a Hill slope of 3.37 for D4(R)-mediated inhibition of Cx43 hemichannel activity. Each bar corresponds to the mean ± SEM of approximately 20 cells from one representative experiment. Experiments were repeated independently three times (N = 3) with similar results.

Experimental screening of the 40 candidate compounds using EtBr uptake assays in Cx43-transfected HeLa cells identified D4(R) as the most potent inhibitor. D4(R) inhibited Cx43 hemichannel activity in a concentration-dependent manner, with an IC_50_ of ∼10 nM and 100% efficacy at concentrations ≥12.5 nM. Under basal conditions, dye uptake was minimal. Exposure to DCFS markedly increased dye uptake, reflecting enhanced hemichannel opening. Treatment with D4(R) suppressed this DCFS-induced uptake in a dose-dependent manner, reducing uptake to near-basal levels at 12.5 nM and above. Carbenoxolone (CBX, 100 µM) was used as a reference hemichannel blocker for assay validation and was not included in the quantitative dose-response analysis shown in Figure 4.

The present study demonstrates potent inhibition of Cx43 hemichannels by D4(R). Previous work from our group reported D4-sensitive connexin hemichannel-related effects involving Cx45 in denervated skeletal muscle, supporting the broader relevance of D4-sensitive connexin hemichannels in pathological muscle remodeling (Cisterna et al., 2020). It is interesting to note that D4(R) did not block Cx39 hemichannels and there was a similar protective effect of D4(R) and Cx43 and Cx45 K.O. in denervated myofibers (Cisterna et al., 2020), suggesting that Cx hemichannels sensitive to D4(R) correspond to the molecular target of this small organic molecule. The nanomolar potency of D4(R) represents a >1,000-fold improvement over existing non-selective blockers such as carbenoxolone and glycyrrhetinic acid, which typically exhibit IC_50_ values in the 10–100 µM range (Verselis and Srinivas, 2013; Manjarrez-Marmolejo and Franco-Pérez, 2016).

Thus, D4(R) clearly inhibited the uptake of a small cationic molecule. Considering that reduced EtBr uptake could result from a reduction in hemichannel pore size and not necessarily from the complete closure of Cx43 hemichannels, we decided to evaluate whether the membrane current of Cx43 hemichannels could be affected by D4(R).

### 3.3 Electrophysiological validation of D4-Mediated Connexin43 hemichannel blockade

To directly assess the effect of D4(R) on hemichannel currents, we performed whole-cell and single-channel patch-clamp recordings of membrane current mediated by Cx43 hemichannels in Hela cell transfectants. Under control conditions, minimal membrane currents were observed. Exposure to DCFS enhanced membrane currents, particularly at positive voltages, consistent with hemichannel activation. Application of the active R-enantiomer, D4(R) (10 nM), markedly reduced these DCFS-induced currents, as evidenced by voltage-ramp recordings and corresponding I-V plots. Single-channel recordings revealed discrete channel-opening events under DCFS, which were abolished by D4(R). Conductance histograms demonstrated that D4(R) decreased both the frequency and amplitude of channel-opening events, supporting a direct inhibitory effect on Cx43 hemichannel activity rather than a reduction in pore size or conductance alone.

**FIGURE 5:**
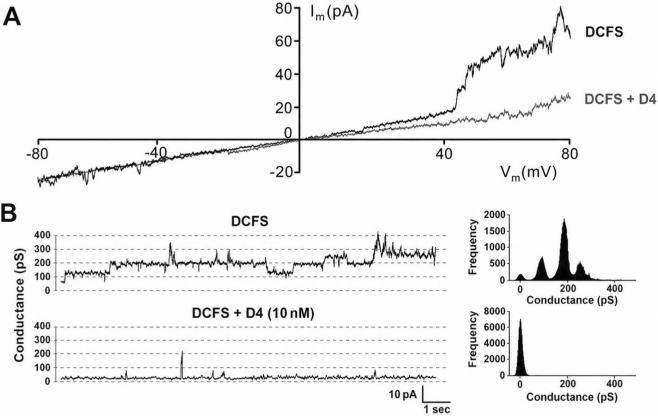
Electrophysiological evaluation of D4 on Cx43 hemichannel membrane currents. **(A)** Voltage ramp was initiated by a transition from −80 mV to +80 mV in HeLa cells transfected with Cx43 exposed first (upper trace) to divalent cation-free solution (DCFS) followed by superfusion with DCFS containing 10 nM D4(R) (lower trace). **(B)** Traces correspond to unitary conductance values of about 220 pS obtained from current event measurements in a Cx43 HeLa cell transfected with Cx43 and exposed to DCFS followed by DCFS plus 10 nM D4(R). Graphs on the right with conductance histograms reveal that D4(R) decreases the frequency of channel-opening events. Representative records from a single cell. N = 3 independent experiments.

**FIGURE 6:**
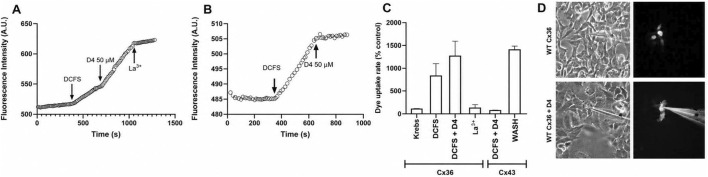
D4 does not block connexin36-hemichannels. Hela cells transfected with mouse connexin36 were used. **(A)** In DAPI uptake experiments, fluorescence intensity increased upon exposure of cells to a divalent cation-free solution (DCFS). Then, cells were sequentially exposed to 50 µM D4(R) [1,000 times more than that required to block dye uptake mediated by Cx43 hemichannels (Figure 4) and 200 µM La^3+^]. **(B)** HeLa cells transfected with mouse Cx43 were used as positive control of hemichannel inhibition by D4(R). In A and B each plotted point corresponds to the average of fluorescence intensity recorded in 20 cells in three independent experiments. **(C)** Summary of fluorescence intensity rate under the indicated conditions, including reversibility upon wash out (Wash) **(D)** Representative fluorescence microscopy images of Lucifer Yellow dye transfer assays in HeLa cells expressing WT Cx36, in the absence or presence of 50 µM D4(R). In A and B, each plotted point corresponds to the mean ± SEM of 20 cells. In C, values correspond to the mean ± SEM from three independent experiments.

Unitary current events of Cx43 hemichannels are frequent in HeLa cells bathed with a saline solution without divalent cations (Ca^2+^ and Mg^2+^) **(** Figure 5). As previously shown (Contreras et al., 2003), the application of a ramp protocol of voltage commands elicited discrete membrane current events that were recorded at positive potentials (>40 mV) and in the same patch (Figure 4). However, the application of 10 nM D4(R) in the same membrane patch completely blocked the discrete membrane current events. Similarly, the application of a rectangular voltage command of +60 mV in cells bathed with DCFS promoted single unitary events of about 220 pS that were completely blocked by 10 nM D4(R) (Figure 6), two lower traces). Evaluations of D4(R) effects on hemichannels formed by other connexins have not been reported yet.

The electrophysiological data confirm that D4(R) acts as a functional hemichannel blocker at nanomolar concentrations, consistent with the dye uptake assays. The complete blockade of discrete hemichannel currents suggests that D4(R) stabilizes a closed conformation or prevents the conformational transitions required for channel opening.

### 3.4 D4(R) does not block Cx36 hemichannels

To assess the isoform selectivity of D4(R), we tested its effect on Cx36 hemichannels, which are predominantly expressed in neurons and play critical roles in electrical synapses (Nagy and Rash, 2000; Bennett and Zukin, 2004). HeLa cells transfected with mouse Cx36 were subjected to DAPI uptake experiments. Exposure to DCFS increased fluorescence intensity, indicating hemichannel opening. Sequential application of D4(R) (50 µM) and La^3+^ (200 µM) revealed that D4(R) did not inhibit Cx36 hemichannel activity, whereas La^3+^ completely blocked dye uptake. This high concentration was intentionally used as a stringent test of sensitivity, since it is approximately 1,000-fold higher than the concentration required to inhibit Cx43 hemichannels, thereby allowing us to determine whether Cx36 hemichannels could be blocked even under exposure to an exaggerated higher concentration. In contrast, HeLa cells transfected with mCx43 showed robust inhibition of dye uptake by D4(R), confirming the positive control. Importantly, after washout out of D4(R) and re-exposure to DCFS, dye uptake increased again, indicating that the inhibitory effect of D4(R) was reversible (Figure 6). These results demonstrate that D4(R) exhibits isoform selectivity, blocking Cx26, Cx32, Cx43, and Cx45 hemichannels but not Cx36 hemichannels and inhibit Cx43 hemichannels in the close or open state.

The lack of Cx36 inhibition is of particular importance, given that Cx36 forms electrical synapses in the brain and retina, and the non-selective blockade of Cx36 could disrupt neural synchrony and network oscillations (Wang and Wu, 2021). The isoform selectivity of D4(R) thus reduces the risk of off-target neurological effects.

### 3.5 D4 spares gap junction channels formed by multiple connexins

**FIGURE 7:**
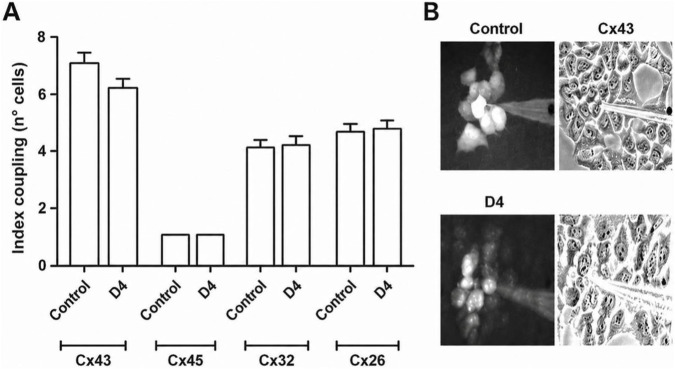
Coupling incidence after compound D4 in gap junctions formed by different connexins. Confluent cultures of HeLa cell transfectants were used. **(A)** Confluent cultures of HeLa cell transfectants expressing mCx43, mCx45, mCx32, or mCx26 were used to evaluate gap junctional coupling by Lucifer yellow microinjection. The fluorescent dye was microinjected in a minimum of 20 cells. Each value corresponds to the mean coupling index ±SEM obtained in three independent experiments, calculated as the average number of coupled cells divided by the number of positive coupling events. No significant difference was obtained in t-tests applied between control and D4(R)-treated cells for each connexin type (t-test, p > 0.05). **(B)** Representative micrographs of Lucifer yellow transfer in HeLa cells expressing Cx43 under control conditions or after treatment with D4(R), showing that D4 did not reduce dye spread through gap junction channels.

A critical question was whether D4(R) selectively blocks hemichannels without affecting gap junction channels formed by the same connexins. We performed Lucifer yellow microinjection assays in confluent cultures of HeLa cells transfected with mCx43, mCx45, mCx32, or mCx36. Coupling incidence, defined as the percentage of injected cells that transferred LY to at least one adjacent cell, was quantified under control conditions and after treatment with D4(R) (10 µM). No significant difference in coupling incidence was observed between control and D4-treated cells for any of the connexin types tested (t-tests, p > 0.05 for all comparisons). These results demonstrate that D4(R) does not inhibit gap junction channels at concentrations that completely block hemichannels (Figure 7).

This gap-junction-sparing property distinguishes D4(R) from classic connexin blockers such as carbenoxolone and glycyrrhetinic acid, which inhibit both hemichannels and gap junctions (Bodendiek and Raman, 2010; Verselis and Srinivas, 2013). The ability to selectively block hemichannels without disrupting gap junction-mediated intercellular communication represents a major advancement in connexin-based channel pharmacology and enables distinctions between the physiological and pathological roles of these two channel types.

### 3.6 D4 exhibits high selectivity over Pannexin1, CALHM1, and TRPV2

**FIGURE 8:**
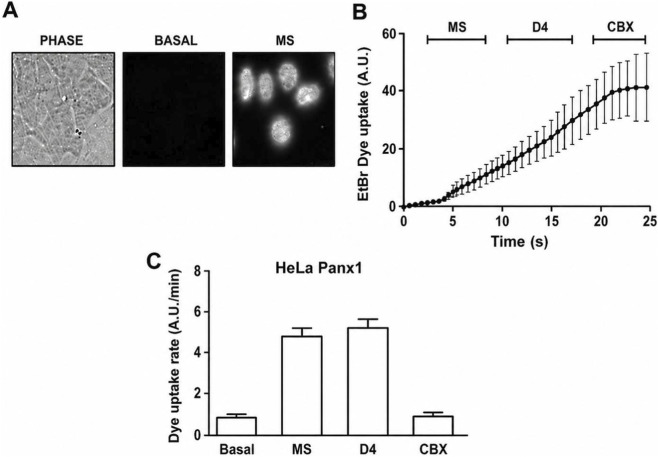
Effect of D4 on Panx1 hemichannel activity in HeLa Panx1-EGFP cells subjected to mechanical stress. **(A)** Representative Ethidium Bromide (EtBr) uptake under control conditions and after mechanical stimulation. Upper panels show a small view of a cell culture observed with bright light (left) and under fluorescent illumination under the control conditions and 5 min after mechanical stretch (MS) stimulation in saline solution containing 5 µM Ethidium Bromide (EtBr). **(B)** EtBr uptake in mechanically stimulated cells treated with 50 µM D4, followed by the sequential addition of 50 µM D4 and 5 µM carbenoxolone (CBX) **(C)** EtBr uptake rate under the indicated experimental conditions. Each plotted point in B corresponds to the mean ± SEM of 20 cells from a representative experiment. Each bar in C corresponds to the mean ± SEM of three independent experiments.

To further characterize D4(R) selectivity, we tested its effects on other large-pore channels that are often inhibited by non-selective connexin blockers. Pannexin1 (Panx1) hemichannels, which mediate ATP release and are activated by mechanical stress, were examined in HeLa Panx1-EGFP cells. Mechanical stimulation markedly increased EtBr uptake, consistent with Panx1 activation. Treatment with D4(R) (100 nM) did not inhibit Panx1 hemichannel activity, whereas carbenoxolone (5 µM) completely blocked uptake **(** Figure 8 **)**. These results indicate that D4(R) does not inhibit Panx1 channels.

**FIGURE 9:**
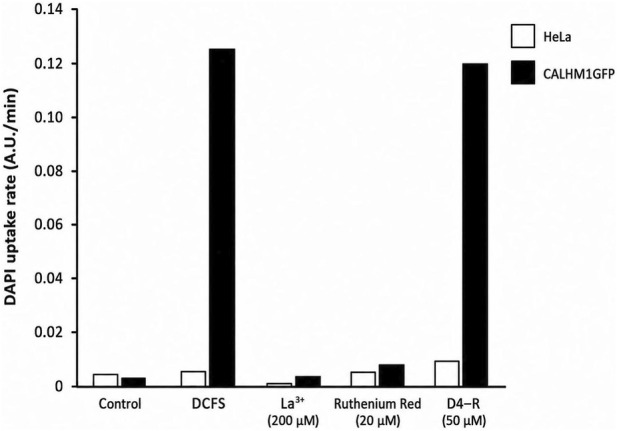
D4 does not inhibit CALHM1 channels. DAPI uptake was evaluated in parental HeLa cells and HeLa cells expressing CALHM1-GFP under the indicated conditions. Removal of extracellular Ca^2+^ and Mg^2+^ (DCFS) markedly increased DAPI uptake in CALHM1-EGFP cells, consistent with CALHM1 channel activation, whereas parental HeLa cells showed minimal uptake under all conditions. The increase in dye uptake rate observed in DCFS was strongly reduced by La^3+^ (20 µM) and ruthenium red (20 µ M), used as CALHM1 channel blockers, but was not reduced by D4 (50 µM). Bars represent average DAPI uptake rates obtained from selected cells/ROIs with comparable CALHM1-EGFP fluorescence intensity within each condition. Individual cells/ROIs were not treated as independent biological replicates, and no SD/SEM or inferential statistical analysis was calculated for this representative panel.

Similarly, CALHM1 channels, which are activated by removal of extracellular Ca^2+^/Mg^2+^ and mediate ATP release in taste cells, were tested in HeLa CALHM1-EGFP cells. Removal of extracellular Ca^2+^/Mg^2+^ markedly increased DAPI uptake in CALHM1-GFP-expressing cells, whereas parental HeLa cells showed minimal uptake (Figure 9). The increase in dye uptake was strongly reduced by La^3+^ (20 µM) and ruthenium red (20 µM), which are established CALHM1 blockers (Ma et al., 2012), but was not affected by D4(R) (50 µM). In this representative CALHM1-EGFP selectivity assay, D4(R) did not reduce the DCFS-induced increase in DAPI uptake, in contrast to La^3+^ and ruthenium red. Cells/ROIs with comparable CALHM1-EGFP fluorescence intensity were selected to reduce variability attributable to differences in expression levels. This panel is presented as a representative selectivity control and was not subjected to inferential statistical analysis.

**FIGURE 10:**
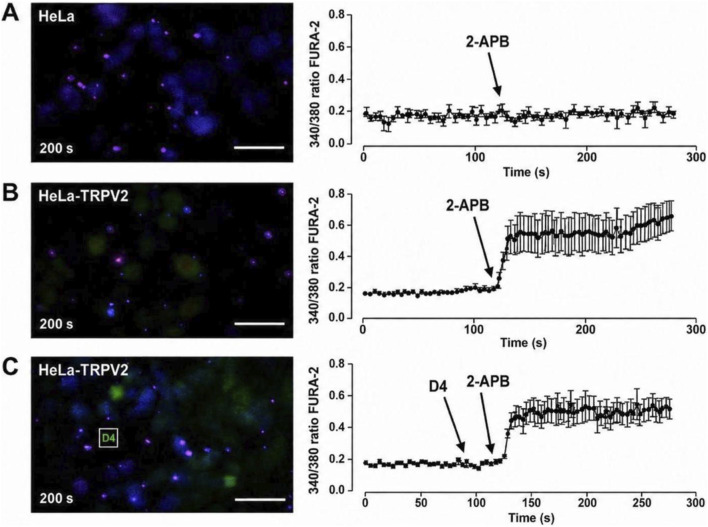
D4 does not inhibit TRPV2 channels expressed in HeLa cells. Left panels: Representative fluorescence images of parental HeLa cells and HeLa cells transfected with TRPV2, showing intracellular Ca^2+^ signals monitored with Fura-2. Right panels: Time course of the 340/380 Fura-2 fluorescence ratio in parental HeLa cells (top) and TRPV2-transfected HeLa cells (middle and bottom, respectively). Adding 2-APB induced little or no change in parental HeLa cells but elicited a marked increase in intracellular Ca^2+^ in TRPV2-transfected cells, consistent with TRPV2 activation. Preapplication of D4(R) (50 µM) did not prevent the 2-APB-induced Ca^2+^ rise. Each plotted point corresponds to the mean ± SEM of 20 cells from a representative experiment (n = 3).

Finally, TRPV2 channels, which are activated by 2-APB and mediate Ca^2+^ influx, were examined in HeLa cells transfected with TRPV2. Intracellular Ca^2+^ signals were monitored using Fura-2. Addition of 2-APB (100 µM), an agonist of TRPV2, induced a marked increase in intracellular Ca^2+^ in TRPV2-transfected cells, consistent with TRPV2 activation, whereas parental HeLa cells showed little or no response (Figure 10). However, pre-application of D4(R) (50 µM) did not prevent 2-APB-induced Ca^2+^ rise (Figure 10), indicating that D4(R) does not inhibit TRPV2 channel activity.

Collectively, these selectivity experiments demonstrate that D4 exhibits high specificity for connexin hemichannels over other large-pore channels, including Panx1, CALHM1, and TRPV2. This selectivity profile is superior to that of classical connexin blockers, which often inhibit multiple channel types.

### 3.7 Synthetic approaches to obtain D4: Stereospecific activity of D4 enantiomers

D4 is a chiral molecule, and we synthesized both the R- and S-enantiomers to assess stereospecific activity. The R-enantiomer exhibited full hemichannel-blocking activity (IC_50_ = 10 nM), whereas the S-enantiomer was inactive at concentrations up to 1 µM. This stereospecific recognition indicates that D4 binds to a defined chiral binding pocket within the hemichannel structure, consistent with the binding mode predicted from virtual screening. The stereospecificity also provides strong evidence that the observed hemichannel blockade is mediated by specific molecular interactions rather than non-specific membrane effects.

**FIGURE 11:**
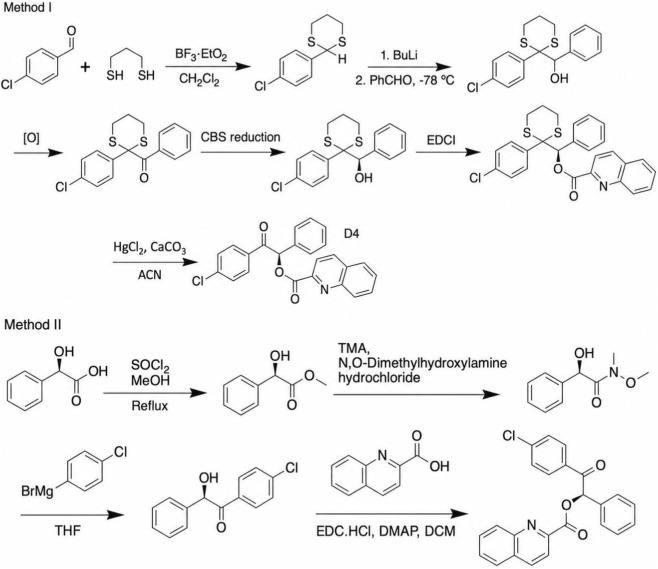
Synthetic routes to the connexin hemichannel inhibitor D4. Method I is a Dithiane-based Umpolung strategy for the construction of the α-acyloxy ketone scaffold. Formation of a 1,3-dithiane from 4-chlorobenzaldehyde enables lithiation with n-BuLi and subsequent nucleophilic addition to benzaldehyde. Oxidation followed by enantioselective CBS reduction affords the chiral secondary alcohol intermediate, which undergoes EDCI-mediated esterification with a heteroaromatic carboxylic acid. Final dithiane deprotection regenerates the carbonyl group, yielding D4. Method II proposes an alternative synthesis *via* a Weinreb amide strategy. An α-hydroxy acid is first converted into the corresponding methyl ester and subsequently transformed into a Weinreb amide. Addition of 4-chlorophenylmagnesium bromide provides the α-hydroxy ketone intermediate, which is then esterified with a heteroaromatic carboxylic acid using EDC·HCl/DMAP to afford the final inhibitor D4.

Two complementary synthetic routes to the connexin hemichannel blocker D4(R) have been disclosed, converging upon a chiral α-acyloxy ketone scaffold decorated with phenyl and p-chlorophenyl substituents (Figure 11). The first route utilizes a classical Umpolung strategy with a 1,3-dithiane acyl anion equivalent. The synthesis starts by protecting 4-chlorobenzaldehyde as a 1,3-dithiane by condensing it with a dithiol under BF_3_·Et_2_O, resulting in a dithiane intermediate that achieves polarity reversal at the original carbonyl carbon. Subsequent lithiation with n-butyllithium at a low temperature produces nucleophilic carbanion, which reacts with benzaldehyde to form a secondary alcohol after carbon–carbon bond formation. This alcohol is oxidized to yield the respective ketone, after which enantioselective CBS reduction yields the desired (R)-configured secondary alcohol, which serves as a key chiral intermediate. Esterification of this alcohol with a heteroaromatic carboxylic acid *via* EDCI-mediated coupling affords an ester intermediate, and the final deprotection of the dithiane results in the regeneration of the carbonyl to achieve entry into the desired α-acyloxy ketone framework corresponding to D4 (Junyu and Huanhuan, 2024).

In comparison, the second synthetic route builds our desired molecular framework with a Weinreb amide strategy that enables stereocontrolled ketone formation. Starting with an α-hydroxy acid, esterification with methanol under reflux generates the corresponding methyl ester, which is subsequently converted into a Weinreb amide using N,O-dimethylhydroxylamine under trimethylaluminum activation. Reaction of this intermediate with 4-chlorophenylmagnesium bromide in THF at a low temperature produces the desired α-hydroxy ketone *via* nucleophilic addition to the Weinreb amide, preventing over-addition and ensuring selective ketone formation. Finally, coupling of the resulting α-hydroxy group with a heteroaromatic carboxylic acid using EDC·HCl and DMAP in dichloromethane affords the esterified product corresponding to D4. While the dithiane-based pathway relies on polarity inversion and asymmetric reduction to introduce chirality, the Weinreb amide approach offers a shorter and more direct strategy for assembling the key ketone intermediate prior to final esterification (Nuñez-Vásquez et al., 2022).

While these synthetic pathways have been described for the synthesis of D4(R), the diversity and depth of organic chemistry provide opportunities for numerous alternative routes. Various synthetic strategies, reagents and catalytic methods can provide access to similar molecular architectures acknowledging that multiple routes have potential utility in making compounds of interest like D4(R).

## 4 Discussion

### 4.1 D4 as a first-in-class gap-junction-sparing hemichannel blocker

The identification of D4(R) represents a significant advancement in connexin pharmacology. For over 2 decades, the field has lacked selective hemichannel blockers that do not inhibit gap junction channels. This gap-junction-sparing property is unprecedented and enables the distinction between the physiological and pathological roles of hemichannels *versus* gap junctions. For example, hemichannel opening in astrocytes and microglia releases ATP and glutamate, driving neuroinflammation, whereas gap junctions coordinate metabolic coupling and electrical synchrony in neuronal networks under physiological conditions, which is reduced during neuroinflammation (Giaume et al., 2021). Selective hemichannel blockade with D4(R) allows these functions to be studied independently. Furthermore, D4(R) exhibits high selectivity for connexin over Panx1, CALHM1, and TRPV2 channels, reducing the risk of off-target effects. This selectivity profile is superior to that of carbenoxolone, which inhibits Panx1 at low micromolar concentrations (Koval et al., 2023), and glycyrrhetinic acid, which can modulate multiple ion channels and transporters (Benfenati et al., 2009; Buckley et al., 2021). Moreover, the stereospecific activity of D4 (R-enantiomer active, S-enantiomer inactive) provides strong additional evidence for a specific molecular interaction with a defined binding pocket, rather than non-specific membrane perturbation.

### 4.2 Structural insights into hemichannel selectivity

The predicted binding mode of D4(R) derived from structure-based virtual screening suggests that the compound binds within a pocket formed by the N-terminal helix (NTH) and transmembrane segments (TM1–TM4). This binding site is consistent with recent cryo-EM studies demonstrating that the NTH plays a critical role in gating and that ligand binding to this region can stabilize closed conformations (Cho et al., 2024; Ding et al., 2024). Specifically, cryo-EM structures of Cx36 revealed that inhibitors such as mefloquine induce displacement of the pore-lining N-terminal helices from a pore-lining (PLN) state into a flexible N-terminal (FN) state, with lipid densities blocking the pore. Similarly, cryo-EM and mutagenesis studies of Cx32 and Cx43 identified two recurring inhibitor sites: A pore-buried site M where mefloquine modifies pore electrostatics, and a site A near the N-terminal gating helix where 2-APB restricts the pore entrance (Lavriha et al., 2025).

The gap-junction-sparing property of D4(R) also indicates a possible preference of the compound binding to the undocked hemichannel conformation rather than the docked gap junction channel conformation. There are also structural differences between hemichannels and gap junctions, including extracellular loop interactions that stabilize the docked state and the possibility that NTH atoms are placed in different regions (Maeda et al., 2009). D4(R) could plausibly bind to a pocket that is either only exposed or stabilized in the hemichannel state, or else docking to hemichannels forming part of gap junction channels present conformational changes that lower D4(D) affinity. Our findings provide experimental validation of computational predictions by our group (Sáez et al., 2025) and others (Comollo, 2018) that the N-terminal helix region serves as a druggable binding site for connexin modulators. While Comollo’s work predicted this site for 2-APB binding, our study demonstrates that structure-based virtual screening targeting this pocket can identify novel, selective hemichannel inhibitors with superior potency and selectivity profiles compared to existing tool compounds. Future structural studies, such as those involving cryo-EM of D4(R)-bound hemichannels and gap junctions, will be essential towards elucidating the molecular basis for this selectivity.

The isoform selectivity of D4(R) (blocking Cx43, Cx45 but not Cx36 and Cx39) also provides insights into structural determinants of inhibitor recognition. Cx36 differs from other connexins in several key residues within the NTH and TM1 regions (Cho et al., 2024). For example, Cx36 has a shorter NTH and distinct amino acid composition compared to Cx43, which may alter the shape or electrostatics of the binding pocket. Recent cryo-EM studies have shown that V37 in Cx32 is a key determinant of mefloquine sensitivity, and the mutation of this residue alters inhibitor potency (Lavriha et al., 2025). Comparative structural analysis of D4(R) binding to different connexin isoforms could reveal the molecular basis for isoform selectivity and guide the design of next-generation inhibitors with tailored isoform profiles.

### 4.3 Mechanistic implications and comparison with recent structural studies

The electrophysiological data demonstrate that D4(R) completely blocks discrete Cx43 hemichannel currents at 10 nM, reducing both the frequency and amplitude of channel-opening events. This suggests that D4(R) stabilizes a closed conformation or prevents the conformational transitions required for channel opening, rather than simply reducing pore conductance. This mechanism is consistent with recent structural insights into connexin gating.

Cryo-EM studies have revealed that N-terminal helix dynamics act as a central gate, with transitions between pore-lining and flexible N-terminal conformations controlling aperture (Brotherton et al., 2024). Posttranslational modifications also modulate gating: Carbamylation-mimetic mutation at K125 in Cx26 biases channels toward a constricted closed state, linking CO_2_ sensing to N-terminal and cytoplasmic loop rearrangements. Similarly, S-nitrosylation of Cx43 at Cys271 is known to activate undocked hemichannels in Duchenne muscular dystrophy cardiomyopathy, promoting arrhythmogenic membrane permeability (Lillo et al., 2019). These studies underscore the importance of NTH positioning and posttranslational modifications in controlling hemichannel activity.

D4(R) may act by stabilizing the NTH in a closed conformation, preventing the conformational transitions that enable channel opening. This mechanism would be analogous to that proposed for mefloquine and 2-APB, which bind to sites M and A, respectively, and restrict NTH movement (Lavriha et al., 2024). Alternatively, D4(R) may induce lipid-mediated pore occlusion, as observed for 1-hexanol and arachidonic acid in Cx36 (Lavriha et al., 2025). Future structural studies, such as cryo-EM of D4(R)-bound hemichannels, will be essential to distinguish between these mechanisms and to define the precise binding mode.

The gap-junction-sparing property of D4(R) also has mechanistic implications. Gap junction channels are formed by docking two hemichannels *via* their extracellular loops, which stabilizes a distinct conformation (Maeda et al., 2009). It is plausible that docking induces conformational changes in the NTH or TM regions that reduce D4(R) affinity, or that the binding pocket is occluded in the docked state. Alternatively, D4(R) may preferentially bind to a hemichannel-specific conformational state that is not accessible in gap junctions. Comparative structural analysis of D4(R) binding to hemichannels and gap junctions will be essential to elucidate this selectivity.

### 4.4 Therapeutic potential and translational outlook

The selective hemichannel-blocking activity of D4(R) positions it as a promising lead compound for therapeutic development. Aberrant hemichannel activity has been implicated in a wide range of diseases, including neuroinflammation, cardiac arrhythmias, skeletal muscle pathology, and skin disorders (Levit and White, 2015; González-Jamett et al., 2022; Araujo et al., 2025; Sáez et al., 2025). Preclinical studies have demonstrated that hemichannel blockade reduces neuroinflammation, spreading depression, and depressive-like behaviors in animal models. For example, a recent study showed that the connexin hemichannel inhibitor D4(R) produces rapid antidepressant-like effects in mice, reducing immobility time in the forced swim test and tail suspension test (Li et al., 2023). Similarly, inhibition of astroglial hemichannels prevents synaptic transmission decline during spreading depression, a phenomenon implicated in migraine and stroke (Tichauer et al., 2024).

In the cardiovascular system, S-nitrosylation of Cx43 at Cys271 activates undocked hemichannels in Duchenne muscular dystrophy cardiomyopathy, promoting arrhythmogenic membrane permeability and myocardial injury (Munoz Camus et al., 2024). Genetic or pharmacological prevention of this modification is cardioprotective in stress models, suggesting that selective hemichannel blockade could be therapeutic in cardiac disease. In skeletal muscle, activation of connexin hemichannels enhances mechanosensitivity and anabolism in disused and aged bone, highlighting context-dependent outcomes of hemichannel modulation (Zhao et al., 2024).

In cancer, connexin hemichannel blockades have shown promise in preclinical glioblastoma models. The monoclonal antibody abEC1.1, which blocks Cx26, Cx30, and Cx32 hemichannels, disrupts glioblastoma progression, suppresses invasiveness, and reduces hyperexcitability (Donati et al., 2025). Similarly, tonabersat, a gap junction inhibitor, enhances temozolomide-mediated cytotoxicity in glioblastoma by disrupting intercellular connectivity through Cx43 inhibition (Schmidt et al., 2025). Moreover, boldine presents an antimitotic action in SVZ NPCs and in GBM cells which may be due, at least in part, to its hemichannel blocking function (Jiménez-Madrona et al., 2023). These studies demonstrate that both hemichannel-blocking and gap-junction-disrupting strategies can be therapeutic when targeted appropriately.

The gap-junction-sparing property of D4(R) is particularly advantageous for therapeutic applications, as it reduces the risk of disrupting normal gap junction-mediated intercellular communication. Gap junctions play essential roles in coordinating electrical activity in the heart and brain, and non-selective blockade could lead to arrhythmias or seizures (Mylvaganam et al., 2014; Žigová et al., 2025). By selectively targeting hemichannels, D4(R) may offer a safer therapeutic profile (Guo et al., 2022; Kiani, 2023).

Several challenges remain before D4(R) can be advanced to clinical development. First, the pharmacokinetic and pharmacodynamic properties of D4(R) *in vivo* need to be characterized, including bioavailability, tissue distribution, metabolism, and half-life. Second, the therapeutic window and potential toxicity need to be assessed in preclinical models. Third, the isoform selectivity of D4(R) (blocking Cx26, Cx32, Cx43, Cx45 but not Cx36) may be advantageous in some contexts but could limit efficacy in diseases where Cx36 hemichannels play a pathological role. Fourth, the stereospecific activity of D4(R) necessitates enantioselective synthesis or chiral resolution to ensure consistent activity. Finally, medicinal chemistry optimization may be required to improve potency, selectivity, or drug-like properties.

Despite these challenges, D4(R) represents a validated starting point for developing next-generation connexin hemichannel therapeutics. The structure-based virtual screening approach used to identify D4(R) can be applied to discover additional chemotypes with improved properties. Furthermore, the structural insights from recent cryo-EM studies provide a rational framework for structure-guided optimization of D4(R) and related compounds (Lee et al., 2023; Brotherton et al., 2024; Cho et al., 2024; Ding et al., 2024; Lavriha et al., 2025).

### 4.5 Limitations and future directions

While this study establishes D4(R) as a first-in-class gap-junction-sparing hemichannel blocker, several limitations should be acknowledged. First, the precise molecular mechanism by which D4(R) blocks hemichannels remains to be fully elucidated. Although the predicted binding mode from virtual screening suggests binding to the N-terminal helix (NTH) and transmembrane regions, direct structural evidence, such as cryo-EM of D4(R)-bound hemichannels, is needed to confirm this hypothesis.

Second, the *in vivo* efficacy of D4(R) has been demonstrated in animal models of neuroinflammation, epilepsy, and depression, but these data are not presented in detail in this manuscript. Future studies should comprehensively characterize the pharmacokinetics, pharmacodynamics, therapeutic efficacy, and safety profile of D4(R) in relevant disease models.

Third, the isoform selectivity of D4(R) remains incompletely defined. The present study demonstrates potent inhibition of Cx43 hemichannels and lack of inhibition of Cx36 hemichannels under the conditions tested. Previous work from our group reported D4-sensitive connexin hemichannel-related effects involving Cx45 in denervated skeletal muscle. However, the selectivity of D4(R) over additional connexin isoforms, including Cx30, Cx37, Cx40, Cx46, and Cx50, has not been systematically evaluated. Given the diversity of connexin isoforms and their tissue-specific expression patterns, comprehensive selectivity profiling will be essential to fully characterize the pharmacological profile of D4(R).

Fourth, the gap-junction-sparing property of D4(R) has been demonstrated using Lucifer yellow microinjection assays, which assess dye transfer between cells. While this is a well-established method for evaluating gap junction function (Veenstra et al., 1994), complementary approaches such as dual whole-cell patch-clamp recordings to directly measure electrical coupling would provide additional confirmation.

Fifth, the effect of D4(R) pre-application before stimulation could not be definitively resolved. Complementary experiments (not shown) showed that D4(R) did not substantially modify baseline fluorescence under Krebs conditions. However, after switching to the activating solution, the fluorescence response rapidly recovered, consistent with the reversible nature of D4(R) inhibition and likely washout or dilution during solution exchange. Therefore, these experiments do not establish whether D4(R) binds to closed channels.

Future directions include: (1) structural determination of D4(R)-bound hemichannels and/or gap junction channels to elucidate the molecular basis for selectivity; (2) comprehensive pharmacokinetic and pharmacodynamic characterization of D4(R) *in vivo*; (3) medicinal chemistry optimization to improve potency, isoform selectivity, and drug-like properties; (4) expansion of the virtual screening strategy to larger chemical libraries, such as ZINC, to identify additional chemotypes with improved potency, selectivity, and drug-like properties; (5) evaluation of D4(R) in additional disease models, including cardiac arrhythmias, skeletal muscle pathology, and skin disorders; (6) development of D4(R) analogs with tailored isoform selectivity profiles; and (7) investigation of combination therapies, such as D4(R) plus standard-of-care treatments, to enhance therapeutic efficacy.

## 5 Conclusion

In the present work, we report the structure-based discovery and experimental validation of D4(R), a first-in-class gap-junction-sparing connexin hemichannel blocker. D4(R) selectively inhibits hemichannels formed by Cx26, Cx32, Cx43, and Cx45 at nM concentrations (IC_50_ = 10 nM) without affecting gap junction channels formed by the same connexins. The compound exhibits high selectivity over other large-pore channels, including Cx30 and Cx36 hemichannels, Panx1 hemichannel, CALHM1, and TRPV2, and demonstrates stereospecific activity (R-enantiomer active, S-enantiomer inactive). Electrophysiological characterization confirms complete blockade of discrete Cx43 hemichannel currents at 10 nM, consistent with stabilization of a closed conformation or prevention of conformational transitions required for channel opening.

The identification of D4(R) addresses a long-standing challenge in connexin pharmacology and provides a validated molecular scaffold for developing next-generation connexin hemichannel therapeutics. The gap-junction-sparing property of D4(R) enables dissection of the distinct physiological and pathological roles of hemichannels *versus* gap junctions and reduces the risk of disrupting normal intercellular communication. The structure-based virtual screening approach used to identify D4(R) can be applied to discover additional chemotypes with improved properties, and recent cryo-EM structural insights provide a rational framework for structure-guided optimization.

D4(R) has demonstrated efficacy in preclinical models of neuroinflammation, epilepsy, and depression, supporting its therapeutic potential. Future studies will focus on comprehensive pharmacokinetic and pharmacodynamic characterization, medicinal chemistry optimization, and evaluation in additional disease models. Collectively, these findings establish D4(R) as a valuable research tool and a promising lead compound for connexin-based therapeutics.

## Data Availability

The raw data supporting the conclusions of this article will be made available by the authors, without undue reservation.
